# Assessment of clinical and radiographic efficiency of manual and pediatric rotary file systems in primary root canal preparation: a randomized controlled clinical trial

**DOI:** 10.1186/s12903-023-03393-1

**Published:** 2023-09-23

**Authors:** Shimaa M. Hadwa, Rehab F. Ghouraba, Ibrahim A. Kabbash, Shaimaa S. EL-Desouky

**Affiliations:** 1 Pediatric Dentistry, Oral Health, and Preventive Dentistry Department, Faculty of Dentistry, Tanta University, Tanta, Egypt; 2 Oral Medicine, periodontology, Oral Diagnosis and Radiology Department, Faculty of Dentistry, Tanta University, Tanta, Egypt; 3 Public Health & Community Medicine Department, Faculty of Medicine, Tanta University, Tanta, Egypt

**Keywords:** Cone-beam computed tomography, Pediatric rotary file, Instrumentation, Obturation

## Abstract

**Introduction:**

The success of primary teeth pulpectomy is strongly reliant on instrumentation techniques and their impact on obturation quality & postoperative pain. Recently, pediatric rotary file systems have been implemented.

**Aim:**

to compare two pediatric rotary file systems (Kedo-S-Square & Fanta AF™ Baby) with manual K-files concerning obturation quality, instrumentation time, and postoperative pain in root canal preparation of primary molars using cone beam computed tomography (CBCT).

**Methods:**

A randomized clinical trial was conducted with the trial registration number (TRN: NCT05619796 and date of registration: (17/11/2022) on sixty primary lower 2nd molars in healthy children aged 4–7 years. Molars were assigned randomly to three groups (n = 20). Group-I and -II were prepared with Kedo-S-Square & Fanta AF™Baby rotary systems respectively while group-III was prepared with a manual K-file. Instrumentation time was recorded using a stopwatch. CBCT was used to assess obturation quality immediately & recorded as optimal, underfilled, or overfilled. Postoperative pain was evaluated at 6, 12, 24, 48 h-time intervals using a four-point pain intensity scale. Statistical analysis was performed for the collected data.

**Results:**

Among the three groups, group-I revealed a greater number of optimally filled teeth (85%) & less instrumentation time (74.75 s) followed by group-II & manual-K file group (p < 0.05). The hand K-file group had significantly more postoperative pain than the two rotary groups (p < 0.05).

**Conclusion:**

the tested rotary file systems resulted in better obturation quality, less instrumentation time, and less postoperative pain compared to manual-K files during primary teeth pulpectomy.

## Key points

Paediatric rotary files allow for effective root canal preparation and reduced chairside time.

CBCT technique’s simplicity with better image quality makes it comfortable for children.

Less postoperative pain is associated with less debris extrusion into peri-radicular tissues using pediatric rotary files.

## Introduction

Primary teeth preservation helps in restricting harmful psychological impacts, assisting in appropriate mastication and speech, and avoiding abnormal oral habits [1]. Pulpectomy is the preferred therapy for pulpal-affected primary teeth because of caries or trauma. Primary teeth root canal therapy is tricky, challenging, and time-consuming due to the problematic tortuous canal anatomy, closeness to the permanent tooth bud, and apparent challenges in behavioral management [2].

Biomechanical preparation is the most crucial step of deciduous teeth pulpectomy, which is primarily prioritized during canal debridement [3]. Conventionally, hand files were used for cleaning and shaping in primary teeth with some disadvantages such as time wastage and iatrogenic error incidences like zipping, lateral perforations, and canal transportation [4].

Barr et al. [5] used Profile rotary files in primary teeth pulpectomy and revealed that the technique is faster, and affordable with consistent results. Also, numerous rotary systems designed primarily for permanent teeth, have been suggested for pediatric endodontics [6]. Since the primary teeth have a ribbon-shaped root anatomy than permanent teeth, these files’ usage in deciduous teeth could produce lateral perforations [7] so, there was an imperative necessity for the development of a unique pediatric rotary system.

In 2017, Jeevanandan [7] introduced the Kedo-S rotary file created especially for primary teeth. Kedo-S files comprised five generations which are Kedo-S [8], Kedo-SG [9], Kedo-SG blue [10], Kedo-S-Square [11], and Kedo-S-Plus [12]. The first generation is made up of three Nickel-titanium (Ni-Ti) files (D1, E1, U1) that have a 12 mm working length and a variable taper to allow effective root canal preparation without over-instrumenting the delicate primary root canal wall [8]. In 2019, a single-file rotary system, Kedo-S-Square file, was invented [7] which consists of two files, A1 for primary anterior teeth & P1 for posterior primary teeth. Kedo-S-Square file has a Ni-Ti heat-treated dual-core with a titanium-oxide coating. Also, it has a unique feature as a variably variable taper design and 16 mm working length with a non-cutting tip [11].

Fanta AF^™^ Baby rotary system, another pediatric rotary file, was produced using Ni-Ti controlled memory (CM)-Wire technology [13] that enables the files to be pre-curved before getting introduced in root canals [14]. These files conform to the root canal shape and don’t totally straighten through curved canal instrumentation. Fanta AF^™^ Baby file has a triangular cross-section with16 mm working length that reduces the stress by minimizing contact between the file & dentin [15].

Obturation quality could be assessed utilizing X-rays, fluid filtering, bacterial leakage, dye infiltration, microscopic examination, radiovisiography, as well as CBCT [16]. CBCT is a three-dimensional(3-D) imaging tool considered an accurate diagnostic radiographical method with low radiation dose due to the ability to select small regions of interest with the simplicity of the technique makes it comfortable to children. Also, it offers more accurate 3-D data about root canal filling volumetric assessment, evaluating the canal’s intricate anatomy, as well as multiple anomalies of the teeth [17]. Better image quality is developed by CBCT with lesser artifacts than conventional computed tomography, which is suitable for endodontic treatment [17].

Few studies have been done to clinically evaluate Kedo-S-Square & Fanta AF™ Baby rotary system. Lakshmanan & Jeevandan [18] compared the obturation quality and instrumentation time of the Kedo-S-Square file, H-file, and K-file for primary molar pulpectomy; it was observed better obturation quality with reduced instrumentation time in Kedo-S-Square group compared to manual instrumentation. Also, Ravindran [19] compared the obturation quality and instrumentation time of hand-k files and Kedo-S-Square files for primary molar pulpectomy; it was reported that the mean instrumentation time of Kedo-S-Square files (72.6 s) was significantly less than that of manual k-files (92.4 s) (p < 0.001) also, a significant improvement in the quality of obturation was observed in kedo-S-Square group. The present study’s purpose was to compare the rotary systems (Kedo-S-Square & Fanta AF™ Baby) with hand-K-files regarding obturation quality, instrumentation time, and postoperative pain in primary root canal preparation using CBCT.

## Materials & methods

### Study setting and ethical consideration

A triple-blinded randomized, prospective controlled clinical study had been performed at the Pediatric Dentistry Department Outpatient Clinic, Faculty of Dentistry, from September to November 2022. The cone beam x-ray was done at the Oral Medicine, Periodontology, Oral Diagnosis & Oral Radiology Department, Faculty of Dentistry, Tanta University. The trial was registered at ClinicalTrials.gov identifier NCT05619796 and the date of registration: (17/11/2022). The study had been approved by the ethical committee (REC), Faculty of Dentistry, Tanta University, code (#R-PED-9-22-2) in accordance with the ethical guidelines outlined in the 1964 Helsinki Declaration and its subsequent revisions. Clinical treatment was started after parents signed written informed consent.

### Eligibility criteria

**Fig. 1:**
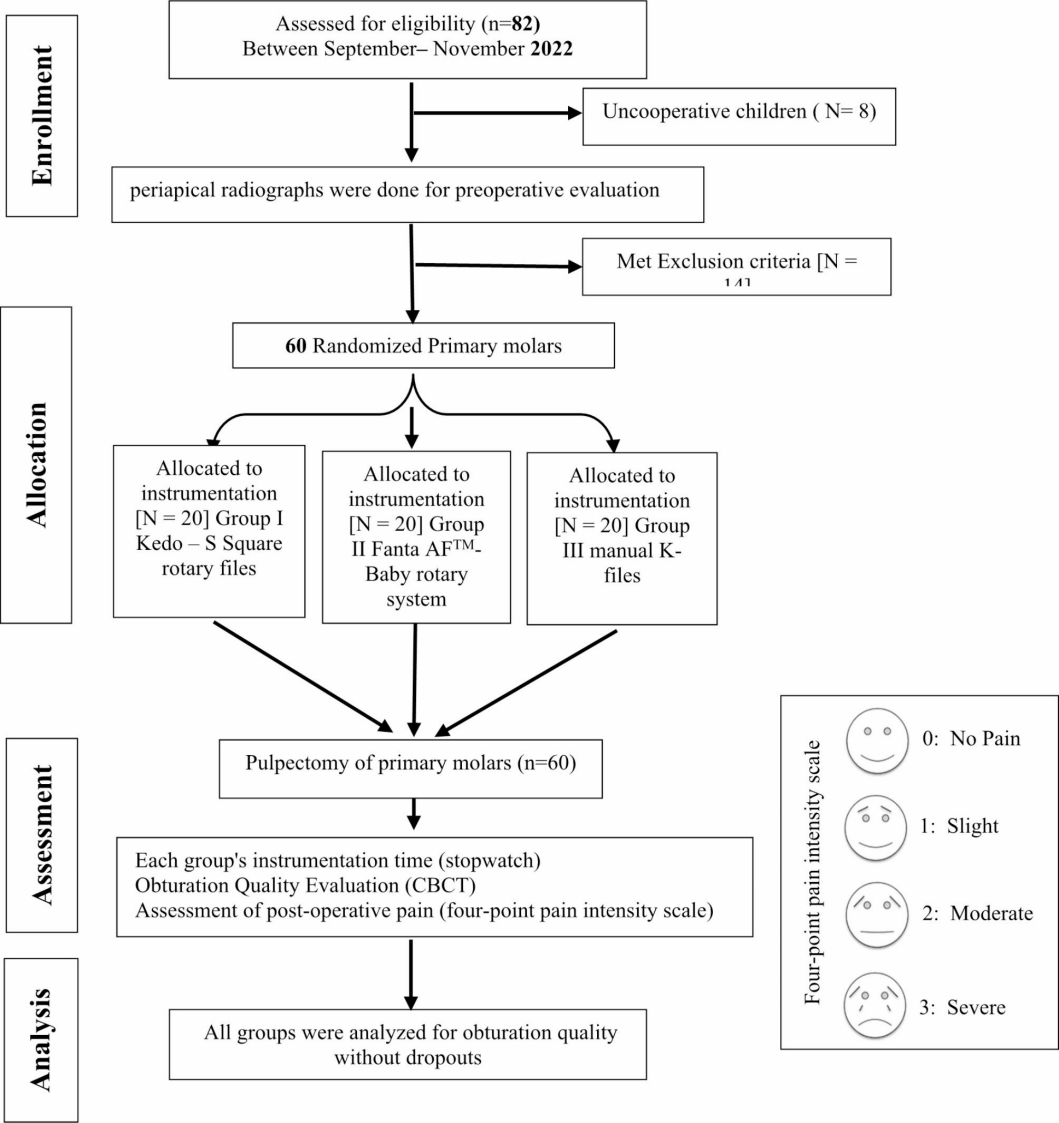
Flowchart explaining the child patient’s randomization and the variables assessed throughout the clinical study

A total of 82 children with an age range of 4-7years who were referred to the CBCT unit of the Oral Medicine, Periodontology, Oral Diagnosis & Oral Radiology Department, Faculty of Dentistry, Tanta University, requiring CBCT imaging for the diagnosis and management of dento-maxillofacial problems, were enrolled and evaluated using the study’s inclusion & exclusion criteria. Inclusion criteria were healthy cooperative children with asymptomatic non-vital mandibular primary second molars with sufficient coronal structure, intact 2/3rd root structure, and no mobility, or external pathological root resorption. Also, the included children should have no history of analgesic use 12 h before the pulpectomy treatment. Uncooperative children or children with systemic illness were excluded from this study. Also, non-restorable primary molars or those with severe mobility, or pathological root resorption were excluded. Accordingly, fourteen patients with non-restorable primary molars, also eight uncooperative patients were excluded. The end-study sample included 60 children who needed CBCT images with no additional radiation exposure for any of the participants. Fig. 1 represents a flow chart that includes enrollment, allocation, assessment, and sample size analysis.

### Sample size calculation and randomization

Using the G-power version 3.1.9 computer program, the sample size was calculated. The study’s power had been set at 80%, and the alpha level was 0.05. The estimated minimum sample size(n) was 15 patients for each group, then it was increased to 20 patients per group. Block randomization was done by an independent researcher to assign sample numbers equally to each group using a1:1:1 allocation ratio and a six-block size by a randomization software (Sealed Envelope Ltd.2021 (https://www.sealedenvelope.com/simple-randomiser/v1/lists)). Then he makes random allocation cards using the computer-generated randomized list that had been preserved in an ambiguous closed envelope after being folded several times. When a patient arrived, the envelope was unlocked. After the treatment ended, the date and patient ID were documented on the envelope.

### Clinical procedure

Single-visit pulpectomy was performed under strict aseptic conditions by a single operator. A perioperative evaluation was done using a digital intraoral photo-simulated phosphorus plate sensor (PSP, Planmeca ProSensor HD, Helsinki, Finland) before starting pulpectomy treatment. The teeth were anesthetized with 2% mepivacaine with 1:20,000 levonordefrin (Alexandria Co., Egypt) and isolated using a rubber dam (Midwest Dental, Texas, USA). Firstly, caries had been removed with sterile no.330 round-carbide burs (Mani Inc., Japan) in a high-speed contra-angle handpiece (NSK, Tokyo, Japan) under copious water cooling and high suction. After gaining the access opening & removing the pulp chamber’s roof, an apex locator (J. Morita MFG Corp., Kyoto, Japan) was used to assess the working length, then affirmed by a periapical radiograph. The working length was determined as one millimeter less than the apex.

The mechanical instrumentation was done using the tested files according to each group. In group-I (*n* = 20): root canals were prepared with rotary P1 Kedo-S-Square files (Reeganz Dental Care Pvt. Ltd. India) at 300 rpm & torque of 2.2 Ncm [20], while in group-II (*n* = 20): root canals were prepared with Fanta AF™ Baby rotary system (Shanghai Fanta Dental Materials, SUNGO Certification Company Limited, London, England) at 350 rpm & torque of 2 Ncm. In the Fanta AF™ Baby system, four files were utilized successively in the following sequence: open-file #17/0.08, #20/0.04 yellow, #25/0.04 red, and #30/0.04 blue [14]. In group-III, the canals were instrumented in sequence using No.15 to 35 manual K-files (Mani, Inc, Japan) using the quarter-turn-pull technique [21]. Rotary files had been utilized with an Endo-Mate DT endodontic motor (NSK, Tokyo, Japan), and Ethylenediaminetetraacetic acid gel (EDTA) 17% (Meta Biomed Co. Ltd, Chungbuk, Korea) was used before instrumentation. 1% sodium hypochlorite (Clorox Co, 10th of Ramadan, Egypt) was used for canal irrigation in-between files after which normal saline irrigation was done. Then, root canals had been dried with paper points (Dentsply Maillefer, Ballaigues, Switzerland) before being packed with Metape x (META Biomed Co, Ltd, Chungbuk, Korea). Intermediate restorative material (reinforced zinc oxide eugenol, IRM® DENTSPLY International, USA) was placed, and then a preformed stainless-steel crown (3 M™ ESPE, USA) was used as a final tooth restoration at the same appointment.

**Fig. 2:**
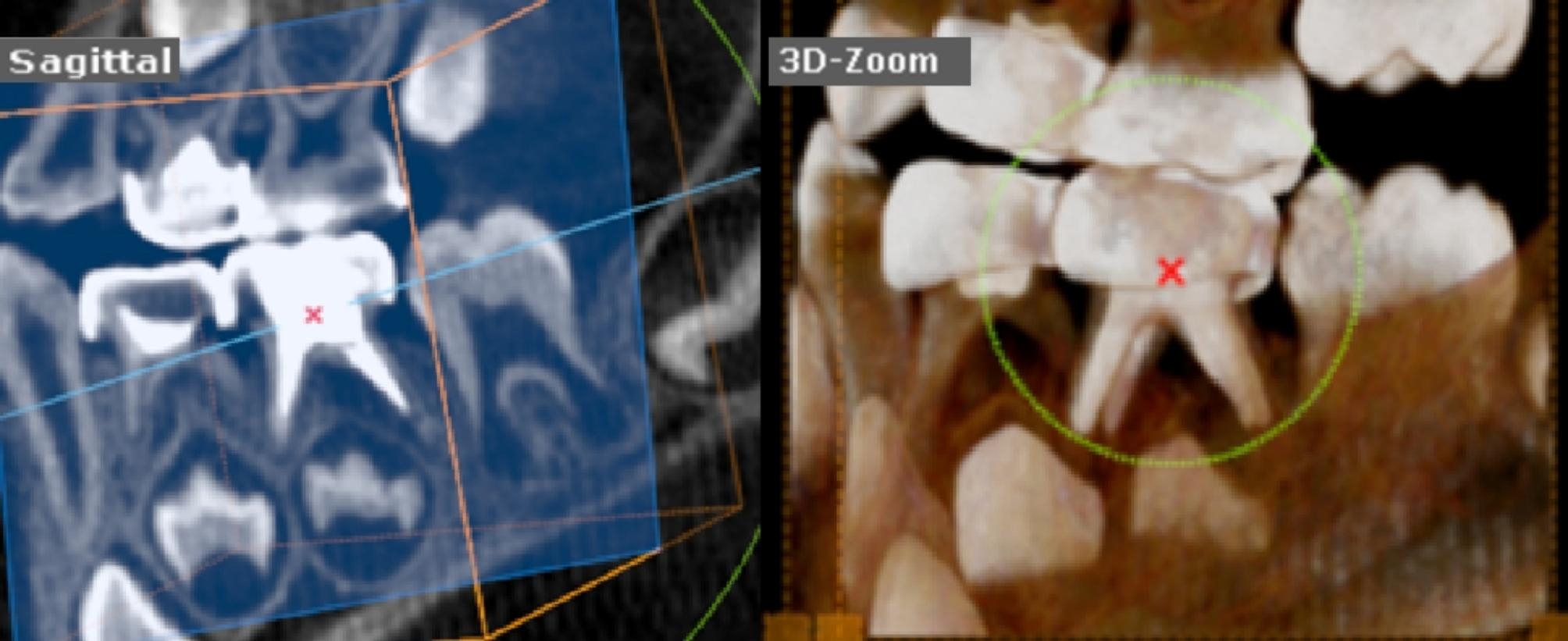
3-D zoom tool had localized the lower left second primary molar with optimal filling obturation in the Kedo-S Square group

**Fig. 3:**
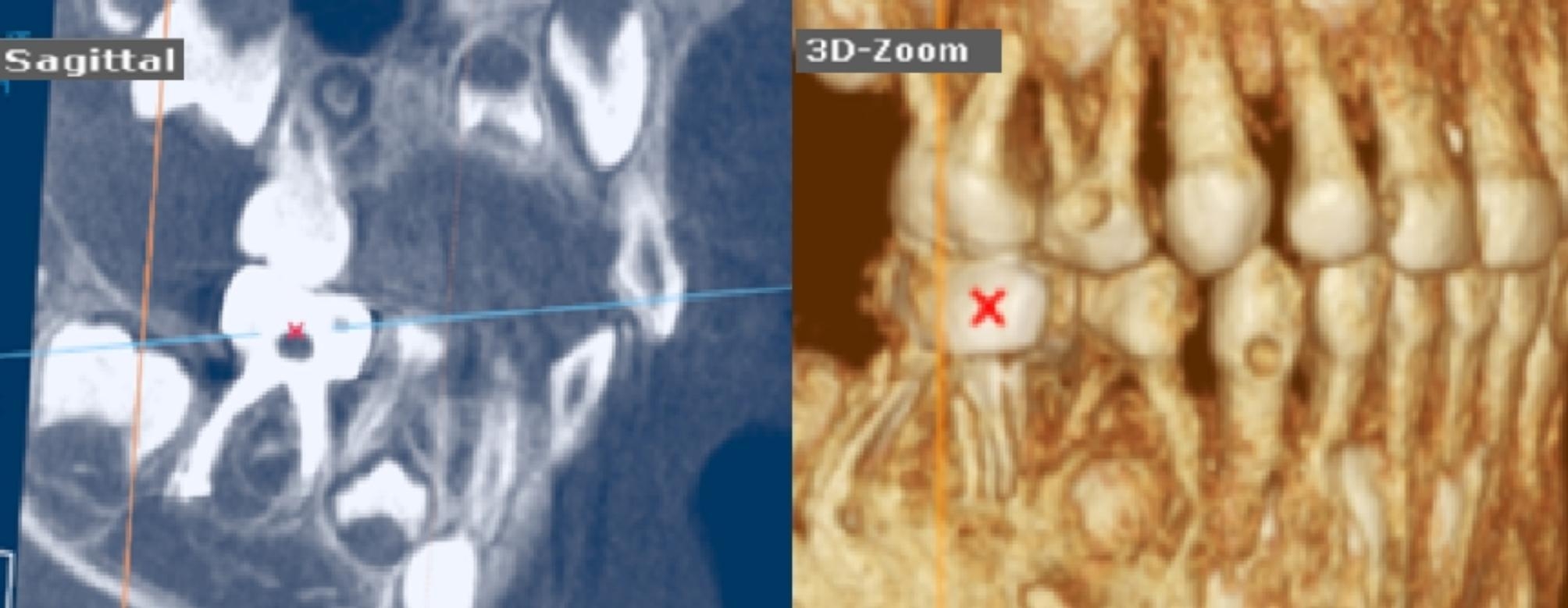
3-D zoom tool had localized the lower right second primary molar with overfilling obturation related to the distal canal in the Fanta AF™ Baby group

**Fig. 4:**
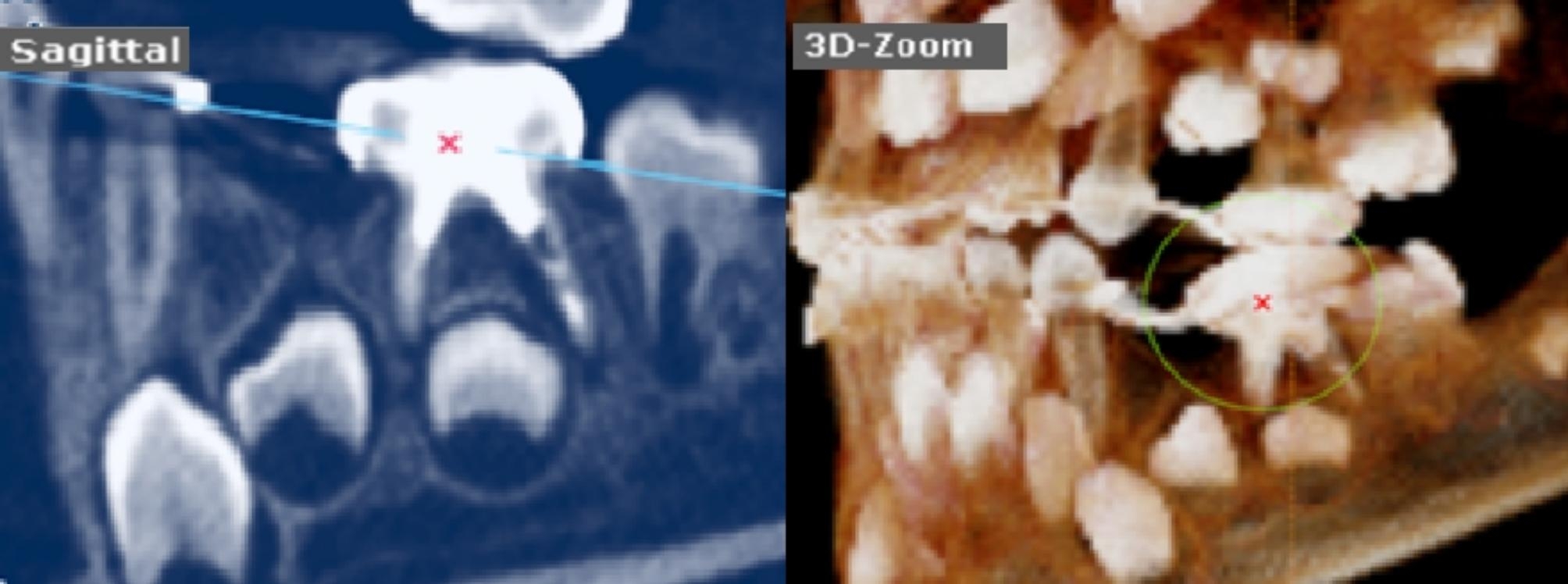
3-D zoom tool had localized the lower left second primary molar with under-filling obturation and void formation in the manual K-file group

Lastly, a post-operative CBCT image (KaVo OP 3D Vision, Kavo Dental, Biberach, Germany) was taken after completing the procedure using quick scan^+^(ultra-low dose) with fixed exposure parameters (90 Kv, 3 mA, and 0.6 mm voxel size) to achieve minimal radiation exposure following the concept as low as reasonably achievable (ALARA) supported by American Dental Association [22]. The CBCT images were obtained by using a 3D module of On Demand Dental software (version 1.0 (build 1.0.10.7462), × 64 Edition, copyright 2004–2017 Cybermed, Korea and license key 670,094,709), the precise location of lower primary molars was detected by using 3D Zoom of the 3D module as shown in Figs. 2, 3, 4 for groups-I, II, and III respectively.

### Instrumentation time assessment

A qualified blinded dental assistant used a stopwatch to record instrumentation time in seconds from instrumentation start to the end of canal preparation. Instrumentation time is the period required to navigate and contour the root canals following access opening and determining the working length.

### Obturation quality assessment

Immediate post-operative CBCT was done for assessing the obturation quality by two trained pediatric dentists, blinded to the instrumentation technique by evaluating Metapex length according to Coll and Sadrian’s [3, 23] criteria as follows Figs. 2, 3, 4:

- Score-1 (Underfilling): Canal filled with metapex that extends more than 2 mm short of apex.
- Score-2 (Optimal filling): Canal filling at the radiographic apex or up to 2 mm short of apex.
- Score-3 (Overfilling): Any canal that has filling beyond the root apex.

In addition, the presence and/or absence of voids were detected as apparent radiolucent areas within the radiopaque metapex in the root canals. Voids were scored as present or absent [24].

### Post-operative pain assessment

Four-point pain intensity scale [25] was utilized to record postoperative pain. According to this scale, pain is divided into four categories: [1] no pain; [2] slight; [3] moderate; and [4] severe. Each participant’s parent was given four notecards to record the postoperative pain at six, twelve, twenty-four, and forty-eight hours after the pulpectomy procedure. Each notecard had four faces as well as a single word to explain each one. Date, child’s name, and a number “I,“ “II,“ or “III” declaring the used file system, were written on each card. A nursing officer who was blinded to the three groups had taught the parents how to use the pain scale. To achieve standardization, the child’s pain score was recorded by the same parent at all follow-up periods. The investigator also recorded the pain scores via a telephonic conversation with the parents to reduce the possibility that they would forget to record the pain at a specific time. Each child got a prescription for an analgesic to be taken in case of severe pain. The children returned to the department two days after the pulpectomy procedure with their completed notecards which were gathered by the nursing officer and given immediately to the blinded statistician.

### Statistical analysis

IBM-SPSS version 19 (Statistical Package for Social Studies) produced by IBM, Illinois, Chicago, USA was used to arrange, tabulate, and statistically analyze the gathered data. Range, mean, and standard deviations for numerical values were computed. Analysis of variance(ANOVA) test and Tukey’s post-hoc analysis were used to compare the instrumentation time among the three groups. To evaluate the obturation quality, the Monte-Carlo-exact test was used. The pain severity readings were dichotomized into binary categories (‘pain’ or ‘no pain’ scores); ‘slight, moderate, or severe pain’ had been all categorized as ‘pain’ [26]. The chi-square test was utilized to evaluate pain intensity over time. When the chi-square test wasn’t appropriate, the Monte-Carlo-exact test was used. The significance level was set at p < 0.05.

## Results

Table 1 showed the demographic allocation of the participants with no significant difference among the three groups regarding gender or age (p > 0.05). The least instrumentation time was observed in the Kedo-S-Square group (74.75 ± 3.99 s) with a significant difference between the three groups (p = 0.0241). The instrumentation time of the manual K-file group (106.2 ± 6.029 s) was significantly longer than the Fanta AF™ Baby group (76.60 ± 3.267 s). Tukey post-hoc analysis affirmed a highly significant difference in instrumentation time between Kedo-S-Square & manual K-file groups and between Fanta AF™ Baby and manual K-file groups (p < 0.001) Table 2.

**Table 1:** Distribution of the study participants by gender and age in years

Groups	Male		Female		Age in years	
	n	%	n	%	**Range**	**Mean ± SD**
**Group I (Kedo-S Square)**	11	55.0	9	45.0	4.5–6.8	5.56 ± 0.75
**Group II (Fanta AF** ^**TM**^ **-Baby)**	12	60.0	8	40.0	4.0-6.8	5.46 ± 0.93
**Group III (Manual-K file)**	9	45.0	11	55.0	4.3-7.0	5.39 ± 0.90
**Total**	32	53.3	28	46.7	4.0–7.0	5.48 ± 0.85

**SD**: Standard Deviation

**For gender distribution: Chi-square**, ***X***^***2***^ = 0.938, p = 0.626

**For age distribution: ANOVA, F** = 0.196, p = 0.823

**Table 2:** Mean instrumentation time (sec)of the three groups

Instrumentation time	Group I (Kedo-S Square)	Group II (Fanta AF^TM^-Baby)	Group III (Manual-K file)	F^∞^	P-value	P_1_-value	P_2_-value	P_3_-value
**Mean ± SD**	74.75 ± 4.00	76.60 ± 3.27	106.2 ± 6.03	295.6	0.024^*^	0.414	< 0.001^*^	< 0.001^*^

SD: Standard Deviation

F^∞^: One-way ANOVA test

*Statistically significant difference at p-value < 0.05

P1-value: Tukey post-hoc of gp I& gp II

P2-value: Tukey post-hoc of gp I & gp III

P3-value: Tukey post-hoc of gp II & gp III

**Fig. 5:**
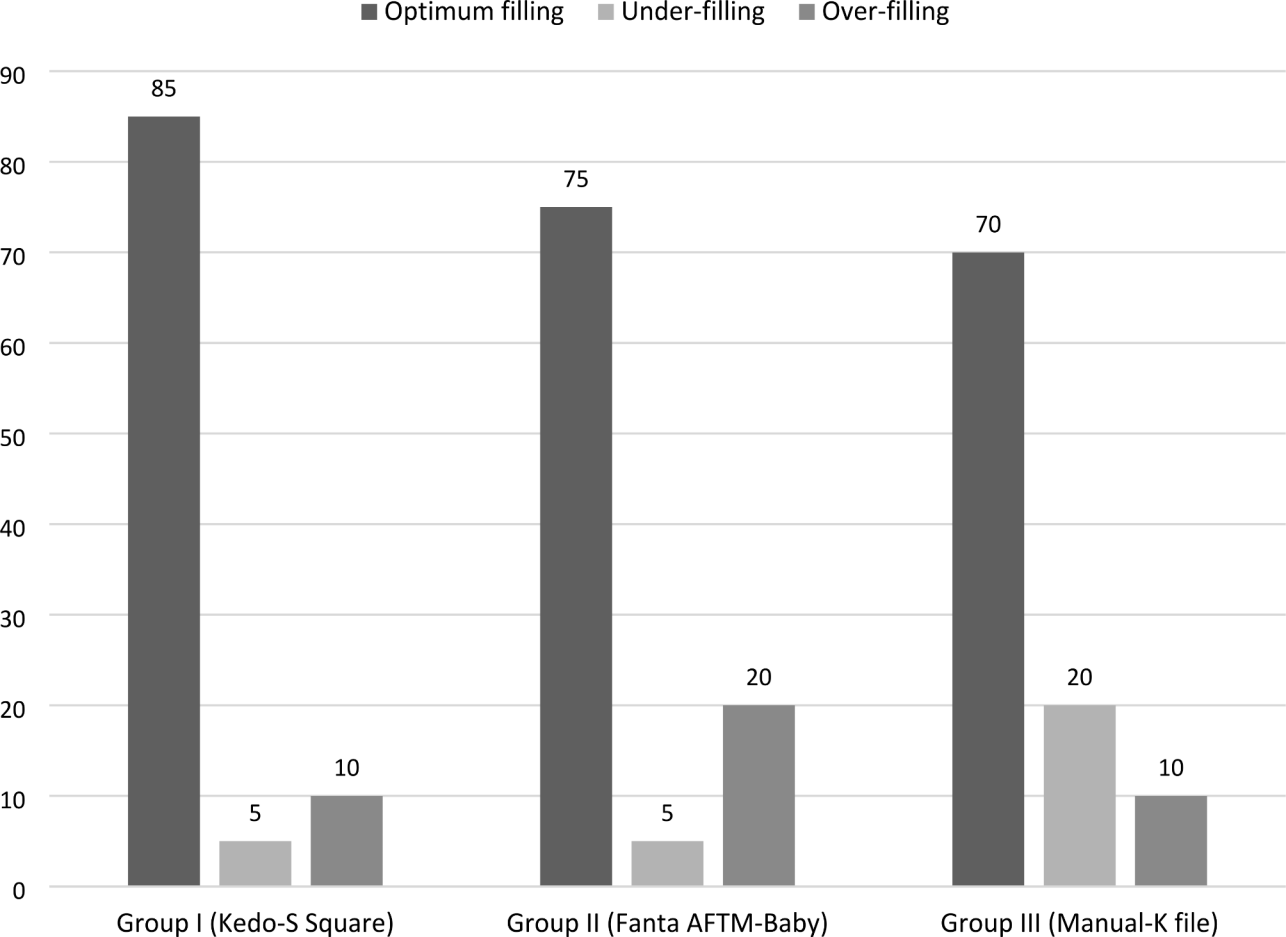
Obturation quality of the three groups

Regarding obturation quality, 85%, 75%, and 70% of teeth instrumented with Kedo-S-Square, Fanta AF™ Baby, and K-files had an optimum filling respectively (Fig. 5). The highest under-filling percentage (20%) was found in the K-file group while the highest over-filling percentage (20%) was observed in the Fanta AF™ Baby group. Using the Monte-Carlo-exact test, there was no statistically significant difference between the rotary groups & k-file group (p = 0.424). Regarding the presence or absence of voids, the highest percentage was found in group-III (manual K-file) (30%), followed by group-I (Kedo-S-Square) (10%), and group-II (Fanta AF™ Baby) (5%) with no statistically significant difference among groups (p = 0.121) Table 3.

**Table 3:** Comparison of presence or absence of voids of the three groups

Groups	Void Presence		Void Absence	
	n	%	n	%
**Group I (Kedo-S-Square)**	2	10.0	18	90.0
**Group II (Fanta AF™** **Baby)**	1	5.0	19	95.0
**Group III (Manual K-file)**	6	30.0	14	70.0

P value of Monte Carlo exact test = 0.121

Throughout the follow-up times, children in the manual K-file group had significantly more postoperative pain than those in the two rotary groups. For all groups, the highest postoperative pain scores were obtained at 6 h, then reduced over time. A statistically significant difference was found between the rotary groups & the manual K-file group (p < 0.05) at all follow-up times Table 4. Regarding each group, Kedo-S-Square & Fanta AF™ Baby groups had no statistically significant difference between different time intervals (p = 0.194, p = 0.261 respectively), while there was a highly significant statistical difference among different time intervals in the manual K-file group (p < 0.001).

**Table 4:** Frequency and percentage of postoperative pain related to different root canal preparation techniques

Post-operative pain	Group I (Kedo-S Square)		Group II (Fanta AF^TM^-Baby)		Group III (Manual-K file)		*X* ^*2*^	P- value
	N	%	n	%	n	%		
**At 6 h**							**20.488**	**< 0.001**
No pain	18	90.0	17	85.0	6	30.0		
Pain	2	10.0	3	15.0	14	70.0		
**At 12 h**							**22.857**	**< 0.001**
No pain	18	90.0	18	90.0	6	30.0		
Pain	2	10.0	2	10.0	14	70.0		
**At 24 h**							**MCET**	**0.013**
No pain	19	95.0	18	90.0	12	60.0		
Pain	1	5.0	2	10.0	8	40.0		
**At 48 h**							**MCET**	**0.004**
No pain	20	100.0	19	95.0	13	65.0		
Pain	0	0.0	1	5.0	7	35.0		
***X*** ^***2***^	4.714		4.000		19.000			
**P**	0.194		0.261		< 0.001			

**MCET**: Monte Carlo Exact test

## Discussion

Successful primary teeth pulpectomy is dependent on the efficiency of biomechanical instrumentation, the obturating material utilized with fewer voids as feasible, as well as the attainment of an excellent hermetic seal [27]. Also, the treatment time is crucial as reduced chairside time increases children’s cooperation for dental treatment, reduces anxiety, and makes the treatment protocol optimal [28]. The instrumentation time was evaluated to ascertain the most trustworthy file system that could be utilized with primary teeth. Another factor affecting the success of primary teeth pulpectomy is the postoperative pain which may be attributed to the fact that extruded materials into peri-radicular tissues stimulate the release of the neuropeptides from nociceptive C-fibres, which include substance P and calcitonin gene-related peptide, accordingly, causing inflammation [29]. CBCT was used to assess the quality of obturation as it has the additional benefit of investigating restricted volumes, allowing every root to be interpreted individually; this was agreed with Naidu et al. [30] & Nezam et al. [31]. While Akhil et al. [1] and Song et al. [32] concluded that CBCT is better compared to periapical radiographs in assessing the canal obturation’s apical extension, however, it is inadequate for assessing the presence of voids. For identifying voids less than 350 μm, digital intraoral techniques outperformed CBCT, while for voids greater than 350 μm, all imaging methods performed similarly [32].

The least instrumentation time had been observed in the Kedo-S-Square group with a significant statistical difference among all groups; this agreed with Silva et al. [4] study which concluded that the rotary Profile 0.04 technique needed significantly less time (3.46 min) for instrumentation than manual K-file (9.06 min). Also, these results are in line with Panchal et al. [33] who found that rotary Kedo-S files compared to hand K-files & H-files needed the least instrumentation time in primary root canal treatment. This may be due to the rotary pediatric file’s adequate working length; this makes it easier to insert and remove the file from the children’s mouth, greatly simplifying treatment for both dentists and the child patients, reducing the need for manual dexterity, increasing operator productivity as well as decreasing the hand fatigue. Also, the lower number of instruments and greater dentine cutting efficacy are contributing factors in less rotary files’ instrumentation time [34]. Kedo-S-square files in the present study needed less time during root canal instrumentation than Fanta AF™ Baby files in primary teeth with no statistically significant difference; this could be credited to the fact of a single file Kedo-S-square system (P1). Conversely, to current study results, Katge et al. [35] showed that manual H files (3.41 min) took less time than Mtwo rotary files (4.81 min) during primary root canal instrumentation. Furthermore, Madan et al. [36] study revealed that more instrumentation time had taken using the ProFiles rotary system (4.97 min in upper roots & 4.30 min in lower roots) compared to lesser time using manual K-files (3.61 min in upper roots & 3.52 min in lower roots). This might be linked to the experience of the operator.

In this study, regarding the obturation quality, the maximum optimal filling had been observed in Kedo-S-Square and Fanta AF™ Baby groups with no significant difference in comparison to the hand K-file group (p = 0.424); this agreed with Jeevanandan & Govindaraju [8] who assessed the obturation quality of Kedo-S system and manual K-file in deciduous molars using peri-apical radiographs and concluded that 77% of Kedo-S group was optimally filled compared to 40% of the hand K-file group with a significant statistical difference (p = 0.015). Also, it was in accordance with Panchal et al. [33] who evaluated the obturation quality of Kedo-S, hand K- & H-files in primary molars using peri-apical radiographs with paralleling technique and revealed that 64% of teeth within KedoS group had optimal obturation compared to 48%, 28% in manual k & H-file groups respectively with a highly statistically significant difference. This could be closely linked to funnel-shaped preparation (wider cervical enlargement & restricted apical preparation) created with the rotary files that enable easier packing of the filling material. Also, the use of Ni-Ti material in rotary file manufacturing increases the file flexibility which helps the file adaptation to the primary canal curvature. The highest under-filling percentage in the present study had been observed in the manual K-file group; this could be referred to less tapered hand files which result in narrow irregular root canals that inhibit the sufficient flow of the filling material. Furthermore, the present study results showed no significant difference in the quality of obturation between the Kedo-S-square & Fanta AF™ Baby groups; this finding is consistent with Govindaraju et al. [2] who observed no significant statistical difference in the quality of obturation between Protaper and Mtwo rotary systems using peri-apical radiographs in deciduous molars (p = 0.125). The highest void percentage was found in the manual K-file, followed by Kedo-S-Square and Fanta AF™ Baby groups; these findings agreed with Naidu et al. [30] who found minimal voids in the Kedo SG Blue group (26.9%), while 34.6% and 38.5% of Pro AF Baby gold and Pedo Flex groups had voids, respectively. Root canal voids can create pathways for leakage leading to bacterial regrowth and infection which raises the possibility of endodontic treatment failure [1].The instrumentation technique, as well as the type, viscosity, and consistency of the paste, obturation technique, and clinician skills, all impact the presence or absence of voids [37].

To decrease the bias during postoperative pain assessment, the following steps were taken. Firstly, only asymptomatic non-vital lower primary second molars had been included in this study as the perception of postoperative pain is strongly linked to preoperative pain, also to equalize the local anesthetic technique as well as the number of root canals. Secondly, only one operator performed the clinical treatment to decrease children’s perception of pain. Lastly, since each child has a different pain threshold, this study used the four-point pain intensity scale which had great reliability & validity, also been frequently used in previous research [20, 38, 39]. The highest postoperative pain scores in the current study were recorded at 6 h in all groups; this could be explained by the distress of the lengthy procedure, and the pressure done by the rubber dam clamp moreover, the anesthesia wears off completely at approximately 6 h after the treatment [29]. By comparing the postoperative pain scores at all follow-up periods, the least percentage was presented in Kedo-S-Square, followed by Fanta AF™ Baby; this could be attributed to less apical debris during root canal instrumentation using rotary files because of the crown down technique, which results in wider coronal preparation and more coronal direction of the debris [39]. Primary teeth are more prone to apical extrusion of debris because of physiological root resorption than permanent teeth. Topçuoğlu et al. [40] evaluated the debris extrusion amount during primary root canal preparation with manual files & three variant rotary files and concluded that ProTaper Next files had been related to little extruded debris apically compared to Mtwo, Revo-S, & manual files (P < 0.05). In the present study, the highest post-operative pain percentage had been found in the manual K-file group at all time intervals; this may be due to a piston-like motion of hand files, resulting in more apical debris. This result is consistent with Topçuoğlu et al. [25] who concluded that the hand K-file group reported significantly more pain than those in the Revo-S group at six, twelve, twenty-four, and forty-eight hours (P < 0.05). Moreover, the present study findings revealed no significant statistical difference among each rotary group at all follow-up times regarding postoperative pain scores; this agreed with Babu et al. [39] who observed no significant statistical difference by comparing Kedo-S & Pedo-Flex rotary file system in primary molars. The presumption that children’s ratings of pain are a reliable indicator of their pain perception during the assessment of postoperative pain could be a limitation of this study. However, there is no other method of confirming that an individual is in pain than believing them.

According to the study’s findings, the Kedo-S-square rotary file system, along with Fanta AF™ Baby files, was more effective than manual files in obtaining an optimum root canal filling with less instrumentation time as well as less postoperative pain. CBCT is ideal for assessing the quality of root canal obturation in three dimensions.

### Clinical relevance

The increased accuracy of CBCT images in evaluating root canal obturation, exploring confined volumes, and facilitating each root to be examined separately has resulted in a more accurate prediction of root canal treatment outcomes.

### Clinical trial registration

“Assessment of Clinical & Radiographic Efficiency of Manual & Pediatric Rotary Systems in Primary Root Canal Preparation: NCT05619796”.

## Data Availability

On reasonable request, the datasets utilized and/or analyzed during the present study are accessible from the corresponding author.

## Abbreviations

CBCT: Cone-beam computed tomography
Ni-Ti: Nickel-titanium
CM: Controlled memory
3-D: Three-dimensional
EDTA: Ethylenediaminetetraacetic acid
ANOVA: Analysis of variance
